# Supplementary material for: The Italian version of the extended Barcelona Music Reward Questionnaire (eBMRQ): a validation study and association with age, gender, and musicianship
Source: PeerJ. 2025 Feb 28;13:e18403. doi: 10.7717/peerj.18403 (PMC11874942; doi:10.7717/peerj.18403)
Supplement: Supplemental Information 1 [file peerj-13-18403-s001.pdf]

Italian version of the extended Barcelona Music Reward Questionnaire (eBMRQ)

Il seguente questionario indaga il tuo rapporto con la musica. Ciascun item del questionario è un’affermazione con la quale puoi essere d’accordo o in disaccordo. Per favore, indica quanto sei in accordo o disaccordo con ciascun item del questionario. Scegli solo una risposta per ogni affermazione. Sii quanto più accurato ed onesto possibile. Non ti preoccupare di essere “coerente” nelle tue risposte.
Per ciascun item, scegli una tra le seguenti opzioni:

[1] - Completamente in disaccordo; [2] - In disaccordo; [3] - Né d'accordo né in disaccordo; [4] - D'accordo; [5] - Completamente d'accordo.

1. Quando condivido della musica con qualcuno, sento una complicità speciale con quella persona.
2. Nel mio tempo libero ascolto poco la musica.
3. Alcune canzoni mi fanno emozionare.
4. La musica mi tiene compagnia quando sono solo.
5. Non mi piace ballare, neanche con la musica che mi piace.
6. A volte mi sento come se fossi un "tutt'uno" con la musica
7. La musica mi fa interagire con altre persone.
8. Cerco informazioni sulla musica che mi piace.
9. Mi emoziono ascoltando certe canzoni.
10. La musica mi tranquillizza e rilassa.
11. La musica mi spinge a ballare.
12. Quando ascolto la musica, posso essere così coinvolto da dimenticare me stesso e ciò che mi circonda.
13. Cerco continuamente novità musicali.
14. Posso piangere quando ascolto melodie che mi piacciono molto.
15. Mi piace cantare e suonare uno strumento con altre persone.
16. La musica mi aiuta a rilassarmi.
17. Non posso fare a meno di canticchiare le canzoni che mi piacciono molto quando le ascolto.
18. Posso sentirmi completamente immerso nella musica, come se il mio stato di coscienza fosse temporaneamente alterato.
19. Ai concerti mi sento in sintonia con gli artisti ed il pubblico.
20. Spendo parecchi soldi per la musica e per cose relazionate alla musica.
21. Mi vengono i brividi quando ascolto una musica che mi piace.
22. La musica mi dà conforto.
23. Quando ascolto una melodia che mi piace molto non posso a fare a meno di muovermi.

24. Quando ascolto della buona musica a volte ho la sensazione di fluttuare nell'aria.
